# Authors’ response to the letter entitled ‘Concerns about the “corporate capture” of The Academy article’

**DOI:** 10.1017/S1368980023001143

**Published:** 2023-09

**Authors:** Angela Carriedo, Ilana Pinsky, Eric Crosbie, Gary Ruskin, Melissa Mialon

**Affiliations:** 1 World Public Health Nutrition Association, Peacehaven, UK; 2 Urban Food Policy Institute, Graduate School of Public Health and Health Policy, City University of New York, New York, USA; 3 School of Community Health Sciences, University of Nevada Reno, Reno, NV, USA; 4 Ozmen Institute for Global Studies, University of Nevada Reno, Reno, NV, USA; 5 US Right to Know, Oakland, CA, USA; 6 Trinity Business School, Trinity College Dublin, Dublin, Ireland

We write in response to the letter entitled ‘Concerns about the corporate capture of The Academy’ published online on 16 March 2023^(1)^. It was written in response to our original research article published in *Public Health Nutrition* on 24 October 2022 about the corporate capture of the Academy of Nutrition and Dietetics (AND)^(2)^.

Our research article provides evidence of AND’s relationships with food, pharmaceutical and agribusiness companies^(3–6)^. We analysed publicly available information accessible to anyone who wants to repeat the exercise^(7)^. We analysed the data using standard scientific methods.

Butler et al. claim that our article ‘has minimum standards of design or research methodology’. The authors are concerned about the contextualisation of the research, accuracy of our reflexivity process, validity and replicability of our study and its rigour. Our research article is written for a public health audience and contextualised under this perspective. Moreover, our original research article provides details on our analysis and data sources, which are comprehensively described in the paper that was peer-reviewed before publication. We undertook an inductive analysis, a well-established qualitative research approach to data analysis, with regular team meetings to discuss analyses and findings. Using documents obtained from freedom of information requests as primary data is a valid data collection technique used in qualitative research^(8)^. We triangulated such data with other publicly accessible documents, including archival AND policies. With the details provided in our article, we are confident that other researchers can replicate our study.

In their letter, Butler et al. point to an author of the original study working for the U.S. Right To Know as having connections to specific organisations. This information is disclosed in our publication and is consistent with the journal’s guidelines. Contrary to what the authors of the letter claim, in scientific articles, it is not a mandatory practice to include information on ‘lived experience, training or roles’. Even if it were, this still would not have changed the substance of our findings.

Butler et al. claimed that our article will have ‘negative implications for the field’. These negative implications are related to our findings: that AND has ties with corporations. We only use the words ‘may’ or ‘might’ when those ties may have influenced the decisions of the AND. In our study, we added evidence to what has been discussed elsewhere and what appears on AND’s website. AND’s members may already be aware of those ties, and some have previously questioned them^(9)^.

It is unclear why the authors of the letter ask the journal to write ‘a statement or position paper on the importance of balancing rigour and ethics in research’ for our article. Every article published in *Public Health Nutrition* follows principles of rigour and ethics in research. These principles are part of the journal’s policies that all authors, including us, adhere to.

In conclusion, we welcome a rigorous scientific debate on our findings. However, calling for the revocation of our article threatens scientific integrity because rigorous scientific standards and processes were followed. We stand by our research and reject any allegations of inaccuracy or other unsupported claims.
